# Perception of Type 2 Diabetes Mellitus (T2DM) patients on diabetes self-care management in Fiji

**DOI:** 10.1371/journal.pone.0304708

**Published:** 2024-05-31

**Authors:** Reshma Kumar, Masoud Mohammadnezhad, Sabiha Khan

**Affiliations:** 1 School of Public Health and Primary Care, Fiji National University, Suva, Fiji; 2 Faculty of Health, Education and Life Science. School of Nursing and Midwifery, Birmingham City University, Birmingham, United Kingdom; 3 Department of Health Education and Behavioral Sciences, Faculty of Public Health, Mahidol University, Nakhon Pathom, Thailand; Bolton Clarke Research Institute, AUSTRALIA

## Abstract

**Introduction:**

The prevalence of diabetes has increased globally where Type 2 Diabetes Mellitus (T2DM) is more common than any other type of diabetes. Self- care management education of diabetes provides skills and information for diabetic patients to effectively perform their own self diabetic self-care for optimum glycemic index control. As T2DM is a growing health issue in Fiji, promoting diabetes self manages among patients is a need, however there is lack of evidence in this regard. Therefore, the aim of this study is to explore the perception of T2DM patients on diabetic self-care management.

**Method:**

This study used a qualitative method among T2DM patients regarding diabetic self-care management in Central Division, Fiji in 2022. This study was conducted in SOPD (Special Out Patient Department) clinics in the three chosen governmental health centers in the central division of Fiji. The study sample inclusion criteria were only T2DM patients, and no other types of diabetes, patients who are 18years and above, patients who are attending clinic at least for more than 6 months, self-identified as Fijian participants of any ethnicity or gender. The study settings were also purposively selected but the study sample was selected using purposive sampling. In depth interview using semi-structured open-ended questionnaires was used to collect data. Thematic analysis was done, followed by reviewing themes, defining and naming them.

**Results:**

Thirty patients participated in this study. Five major themes emerged from the in-depth interview including; patient factors that affect diabetes self-care management, behavior and attitude towards T2DM self-care management, health services delivery, challenges and barriers faced by patients to perform diabetes self-care management, and recommendations to improve patient self-care management. Patients in this study have good knowledge about T2DM and the self-care management they have to perform. It is the patients’ attitude and behavior towards T2DM self-care management that affects patients to perform self-care management. The study also showed patients have gained good knowledge from Health Care Workers (HCW). Socio-economic and psychological status also played a vital part in patients’ self-care management. Apart from challenges, there were opportunities to learn the difficulties patients face in order to perform self-care management.

**Conclusion:**

The results of this study revealed a combination of individual, cultural, and health systematic related factors as the mots influencer of diabetes self-management among patients in Fiji. Patients have to take ownership of their own health in order to improve their blood sugar reading and reduce complication of diabetes. Tailored interventions that consider patients’ belief and address potential challenges would be useful. A lot is needed in terms of upgrading facilities for the comfort of patients and need to collaborate more with other multidisciplinary team and stakeholders.

## Introduction

The World Health Organization (WHO) recognizers diabetes mellitus as one of the top four Non-Communicable Diseases (NCDs) [1]. Type 2 Diabetes Mellitus (T2DM) is chronic, metabolic disease that occurs when the insulin secretion by the pancreatic is defective or when the insulin-sensitive tissue is not able to respond to insulin resulting in high glycemic index [2]. The prevalence of diabetes has increased globally where T2DM is more common than any other type of diabetes [3]. The Global Burden of Disease (GBD), estimated in year 2017, there were 462 million people around the world suffering from T2DM [4]. WHO has predicted by 2030, diabetes will be the world’s top 7 leading cause of death and 77.6% of diabetes cases will be in developing countries [5]. The risk factors for T2DM are age, physical inactivity, overweight and obesity. Smoking and increase body fat are also risk factors for T2DM [6]. The dietary pattern is strongly associated with T2DM where healthy diet improves the glycemic index, whereas western diet including red meat, fast food, and refined grains increases the risk of T2DM [5]. The compendium of studies in United States (US) show diabetes has caused increase in the disability, visits to physician, premature mortality, hospitalization, and costs that amounts almost $37 billion in the 2017 due to lost productivity and health care cost [7]. Diabetes is now the leading cause of complications to the eyes causing blindness, amputations and kidney failure [8].

Self-care management education of diabetes provides skills and information for diabetic patients to effectively perform their own self diabetic self-care management for optimum glycemic index control [9]. Diabetes self-care management is an evolutionary process where awareness or development of knowledge is through complex social context. It is a must for individuals suffering from T2DM to perform self-care management activities in their day to day lives such as regular exercising, appropriate diet, monitoring their own blood glucose level, self-medication administration and foot care [10]. Even though, living with T2DM has a huge impact in every aspect of patients’ life, the quality of living can be normal if patients perform proper self-care management by modifying to healthy eating habits, regular exercise, taking the prescribed medications and self-monitoring blood sugar level [11]. Good adherence to medications can improve the blood glucose level and delay the complications of diabetes [8]. Several studies have shown that despite knowing the benefits of diabetes self-care management, only 15.1% of T2DM patients adhered to self-care management [11]. In the US, half of the patients are in poor control of the glycemic control due to noncompliance to medications and poor adherence to physical activity [8]. All T2DM patients need good education and support on diabetic self-care management in order to improve their quality of life.

The Western Pacific region had 138.2 million individuals with T2DM in year 2013 with [3]. In Fiji, 1 in every 3^rd^ person is diagnosed with diabetes which accounts 30% of the general population where T2DM is more common than any other type of diabetes [12]. The surgeons in Fiji perform about 8 to 10 surgical operations related to T2DM in which at least 1 operation will be amputating the lower limps [13]. The 2002 STEPwise approach to non-communicable risk factor surveillance (STEPS) survey, showed diabetes is common in both the ethnic groups; i-Taukei and Fijian of Indian descent (Ministry of Health and Medical Services, 2018). Fijian of Indian descent population has 11.5% higher incidence of diabetes then the i-Taukei population. The prevalence of diabetes in Fiji increased from 14% in 2002 to 16% in 2011. The 2011 STEP survey showed an increase in the blood glucose level and obesity [14].

T2DM has been the leading cause of death in most countries and is predicated to increase in the coming future due to the socioeconomic development in developing countries [3]. The incidence rate of diabetes globally is 7.4% per 1000 between the ages 20–79 years [15]. When diabetes is not controlled, severe complication begins which affects the quality of life in patients. The treatment cost of type 2 diabetes is 2–4 times more than the ordinary individual where diabetes cost higher than any other disease [5]. The outpatient record 2013–2018 showed that 176,000 patients were only treated for diabetes, with total of $406millions dollars estimated burden on Fijian society due to NCDs, whereby the Fiji government called this burden as a crisis. The burden is not only on the government, but it affects the individual (physically and psychologically), family, social life and work life [16]. T2DM patients receive all the care, information and medical treatment in the clinics but still the severity of disease worsens, thus it becomes important to know why the severity gets worse. It becomes vital to know if the patients are performing diabetic self-care management. Individuals with diabetes gets depressed which affects an individual’s problem-solving skills which than results poor self-care management [17].

What is already known in the study is the diabetic self-care management is performed through medication, diet and exercise but still there is no improvement in glycemic index. Self-care management has been explained to diabetic patients, but it is not clear how often exercise, healthy diet and medication is to be incorporated daily [8]. Through the study, we will be able to know patients’ knowledge about T2DM and medication, the attitudes towards self-care management, the challenges faced by the diabetic patients to adhere to diabetic self-care management.

In Fiji, there is a need to get T2DM patients personal experience in self-care management as previous literature doesn’t show evidence in this regard. The previous findings and data suggest the presence of large gap between current practice and the recommended guideline where both need and opportunity to enhance the rates of diabetic self-care management and support among patients to perform diabetes self-care management. Therefore, this study aimed to explore the perceptions of T2DM patients on diabetic self-care management in Suva, Fiji. This research will aim to gather as much information as possible to help MOHMS and policy makers to tailor management where diabetic patient can adhere to diabetes self-care management. It will also benefit T2DM patients to improve their quality of life. The aim of this article is to explore the perception of T2DM patients on diabetic self-care management.

## Methodology

### Design and setting

This study used a descriptive qualitative method that focuses on perception of T2DM patients on diabetes self-care management in Central Division, Suva, Fiji in 2022. The qualitative study methods provided comprehensive answers from patients which also explores the understanding of participants’ behavior; how and why participants in the study respond to self-care management. This study method is very flexible in nature of exploration which has advantages to the researcher investigate knowledge, barriers, attitude, feelings and perception to respond [18].

This study was conducted in Special OutPatient Department (SOPD) clinics in the three purposively chosen governmental health centers in the central division of Fiji called; Makoi Health Center (MHC), Valelevu Health Center (VHC), and Samabula Health Center (SHC). The three health centers were chosen randomly from among seven health centers in Central area to give a mix of urban and peri-urban setting to the study. The health centers have a SOPD which conducts clinic for NCDs such as diabetes, hypertension, stroke, and cardiac cases.

### Study sample

The study population included T2DM patients from three different selected health centers. The population that meets the inclusion/exclusion criteria were selected to be in the study sample. The participants who consented were selected to be part of the study. Inclusion criteria were only T2DM patients, and no other types of diabetes, patients who are 18years and above, patients who are attending clinic at least for more than 6 months, self-identified as Fijian participants of any ethnicity or gender. The exclusion criteria for patients were patients who are not willing to take part and those who have medical condition and are not able to participate in the study.

In the study, participants were chosen using purposive sampling. The sample size for the patients was 30 patients (10 from each health center). Face to face In-depth interview was conducted among all the selected 30 T2DM patients until data saturation was reached, and there was no new information elicited regarding perception, knowledge, attitude towards diabetes self-care management [19].

### Data collection tool

A semi-structured open-ended questionnaire was used to guide interview patients. Semi-structured in-depth interview is known to be the sole source of information in qualitative research where researcher can go deep and highlight on individuals’ personal issues [20]. The questionnaire was designed according to previous studies as well as the research questions. The questionnaire had 2 sections (Annex 1.1). Section 1 had demographic data (7 questions), section 2 included 19 open ended questions to understand their perceptions towards T2DM and self-care management.

The questionnaire was tested in a pilot study among 4 patients who met the criteria to check for validity and to check if the questions were clear to understand. The content validity involved 3 experts to verify the semi-structured open-ended questionnaire.

### Study procedure

Permission was taken from Permanent Sectary of Ministry of Health and Medical Services and Sub divisional medical officer to conduct study in health centers. Once the permission was granted, permission was taken from Medical Officer in-charge at Makoi Health Center, Valelevu Health Center, and Samabula Health Center for the study to be conducted at their health facilities. The recruitment period for this study was from the 1st to 22nd of August, 2022. The flyers and information sheet were printed and distributed 2 weeks prior to the commencement of data collection to Makoi Health Center, Valelevu Health Center, and Samabula Health Center. Staff working in SOPD clinic were explained about study and request was made to inform on the clinic day that has appointment dates for patients meeting the inclusion criteria. Only participants that agreed and consented to participate were interviewed.

The patients were provided with an information sheet prior to the interview. Those who agreed to participate in the study were given a written consent form prior to the interview. For the patients both information sheet and consent form were in 3 languages (Indo Fijian, I-taukei and English). For I-taukei participants, bilingual translator was arranged prior to the interview day.

Interviews were conducted by a trained interviewer in a quiet room that is suitable and comfortable for both participants and interviewer. In depth interview toke around 30minutes. All the interviews were recorded by a digital recorder.

### Data management and analysis

As soon as the interview was completed, the data was transcribed by the principal investigator into Microsoft word. The transcript was checked by listening to the records and read repeatedly by principal researcher in order to identify typo errors, or if they missed out and hearing the voice recorder and correct before transcribing the others. Manual thematic analysis was used to analyze the data. Thematic analysis was done by closely reading and interpreting the data to identify common themes patterns and ideas coming up repeatedly, followed by reviewing themes, defining and naming them [21]. The steps followed were; identify themes, once identified, coding was done in order to refine and develop the information obtained; the themes were searched and reviewed. When reviewing themes, the researcher paused and saw the “fit” in code and if the answers are clear and distinct; in case the themes are not clear, phase was restarted; data extracts were compelled.

### Study rigor

For the study process to be trustworthy, Lincoln and Guba [22], proposed that any research should satisfy the four–dimensional criteria. The four–dimensional criteria (credibility, dependability, confirmability and transferability) was implemented to assess the quality of the research. The credibility ensured that the study measured what was intended and was a true reflection of the participants in the society [23, 24]. During the study, credibility was achieved by using the inclusion criteria as self-identified as Fijian participants of any ethnicity or gender. Transferability ensured that the ability of the findings could be transferred to other setting or research [24]. This research was just conducted in the three selected health centers with a small sample size; however, this research can be applied to a larger sample and other health centers in other divisions as well. Dependability ensured that the process was described in sufficient detail so that another researcher could repeat the work [23, 24]. Dependability was achieved by stating all process with enough detail for other researchers. Confirmability is the process to extend the confidence that the results would be confirmed or corroborated by other researchers [23, 24]. The researchers at Fiji National University checked and rechecked throughout data collection and analysis to ensure results were repeatable by other researchers.

### Ethical consideration

Ethical approval was obtained from College Human Health Research Ethics Committee (CHHREC) of Fiji National University (FNU) and the Fiji National Health Research and Ethics Review (FNHRERC). All the participants were informed about the study procedure using information sheet and were asked to sign a written consent form before participating in this study. All participants’ confidentiality was maintained at all times. Participants names were not used when the data was collected and also the quotes were used in the results section, (instead a unique code was used). All participants’ confidentiality was always maintained. The recorded interview and transcribed scripts were only accessible to the principal investigator which was kept in a laptop with password protection. The raw data was locked in the locker and under no circumstances shall it be disclosed to any other individual.

Freedom was given to the participants if they wish to withdraw during the interview and are not willing to participate. They were able to choose an information sheet and consent form in their preferred language. All the data that was gathered was securely stored in a protected laptop with a secret password.

## Results

### General characteristics of participants

A total of 30 participants were interviewed for the study. The majority of the participants age were above 60years. More than half of the participants were male, one third of the participants were I-Taukei, and other two third were Fijian of Indian Descent. Only 3.3% of the participants never went to school. The majority of the participants were married and also the majority of the participants were unemployed. 50% of the participants earn <$500. (Table 1)

**Table 1:** Summary of patients’ characteristics (n-30).

Variables	Frequency	Percentage
**Age group**		
<40	1	3.3
40–49	2	6.6
50–59	9	30
≥ 60	18	60
**Gender**		
Male	16	53.3
Female	14	46.7
**Ethnicity**		
I-Taukei	11	36.7
Fijian of Indian decent	19	63.3
**Religion**		
Christianity	11	36.7
Hinduism	15	50
Muslims	4	13.3
**Education**		
Never	1	3.3
Primary school	14	46.6
Secondary school	9	30
Tertiary	6	20
**Marital status**		
Single	2	6.67
Married	18	60
Widow	10	33.3
**Employment status**		
Employed	4	13.3
Non-employed	26	86.6
**Monthly income ($)**		
<500	15	50
500–1000	10	33.3
>1000	5	16.6

#### Themes identified

By identifying similar codes that gave the same meaning, sub-themes were generated. Similar sub-themes then were grouped under a main concept that was called theme. The responses from the participants emerged to five major themes: patient factors that affect diabetes self-care management, behavior and attitude to T2DM self-care management, health services delivery, challenges and barriers faced by patients to perform diabetes self-care management, and recommendations to improve patient self-care management. Each participant was given a unique identification number. Participants were given numbers ranging from M1 to M30. The themes and sub-themes are presented in Table 2.

**Table 2:** Summary on themes, sub-themes and codes identified among patients.

Themes	Sub-themes	Code
Patient factors that affect diabetes self-care management	Knowledge on diabetes self-care management	To take medication always, no sugar intake, I know how to look after myself, I know about my disease and how to manage, I google about diabetes care.
	Psychological factors affecting to perform diabetes self-care management	Worried about diabetes, was on denial stage, I did not expect to have diabetes, stressed to know I am diabetic, disheartened.
Behavior and attitude to diabetes self-care management	Reinforcing factors for lifestyle and physical activity	Lot of functions to attend, eat a lot during festive time, grog consumption during social gathering, alcohol, I follow proper diet, walk only, when ever feel like, House work, Never, no time, feel tired easily, go to Zumba class, go to gym sometimes.
	Monitoring of blood sugar level as self-care management	It is very painful to check sugar every day, don’t take CBG reading daily, only do sugar test when I have dizziness or headache, only check when I am very thirsty.
	Compliance to medication as self-care management.	Always take on time, never miss, take all the medications as prescribed, sometimes I no.
	Use of Herbal medication	I take herbal drinks, take leaves when sugar level is high.
Health service delivery	Service provided at health facilities	Very good service, no rush, clear explanation, no medications provided at times, have to wait for long at times
	Staff attitude influencing self-care management	Very good staffs, explanation is very clear, soft spoken, polite, rude at times. Too harsh sometimes.
	Attitudes toward conditions/health care environment at health facilities.	Overcrowded area to sit, feel hot and stressed when have to wait for long.
Challenges and barriers faced by patients to perform diabetes self-care management.	Socioeconomic status affecting self-care management.	Less money to buy proper food, not enough space to plant my own food or sell, have to support other family members, do not full time job, have to eat whatever is available, have other needs to look after.
	Family support influencing self-care management	No family supports, have to cook my own food, I have to work to buy my food, I eat whatever is prepared at home by daughter-in-law, no choice but to eat whatever is available.
	Schedule for clinic date when working	Have to take time out from work, less paid when I take hours off for clinic, cannot come due to work.
Recommendations to improve patient self-care management	Flexible hours for working patients	Not accessible during working hours, other health department should be also scheduled.
	Quality of life	Include family therapy about diet, have home visits by dietician to talk to other family members about meal preparation, special place in community to exercise.
	Expansion of waiting rooms to create space and prevent congestion as patients wait for services.	More space is needed, more sitting chairs and benches needed to sit, increase the space, relocate clinic waiting area.

#### Patient factors that affect diabetes self-care management

There were two subthemes found under patient factors: knowledge on diabetes self-care management and psychological factors.

*Knowledge on diabetes self-care management*. For all the participants, they had knowledge on diabetes self-care management. Most of the participants mentioned diabetic diet, exercise, medications on time, foot care, sugar check and meal times (n = 30).

“*Yes*, *I know about diabetes and how to take care of myself*. *I don’t have to take sugar and sweet foods*, *I have to exercise*, *I have to take all my medications on time and drink plenty of water*.*” (50-year-old Male Patient M1)*

One participant said, with assistance of grandchildren, he is able to know about diabetes self-care management through google search and see videos to help perform self-care management.

“*I know how to take care of myself*. *My grandchildren help me watch videos on Google on how to take care of myself*. *Now I also know how to use google and look for more diabetes care*, *and how I can do it myself*.*” (62-year-old Female Patient M10)*

*Psychological factors affecting to perform self-care management*. The participants were asked how they felt when they were first told they have type 2 diabetes and what are their thoughts now regarding diabetes. Most of the participants were worried about the disease when they came to know about it.

“*I was so worried about the disease when I was first told and even now*, *I am worried when my blood sugar level is high*.*” (45-year-old Female Patient M2)*

There were few patients who were at denial stage when they were told about the disease and did not accept to perform diabetes self-care management.

*“When I was first told that I have diabetes*, *I did not believe*, *I did not trust the doctor*, *I when to other doctors to check what is wrong with me*. *When the private doctor also said my that I have diabetes then I believed*. *I got so worried*, *I did not eat for weeks*, *I lost interest in doing my day to day activities*. *Know I fully accept my disease and I try my best to keep my sugar level down*.*” (72-year-old Male Patient M14)*

**Behavior and attitude towards diabetes self-care management.** There were four sub-themes identified under behavior and attitude of patients towards diabetes self-care management: reinforcing factors for lifestyle and physical activity, monitoring of sugar level, compliance to medication and herbal medications.

*Reinforcing factors for lifestyle and physical activity*. Some of the patients have lifestyle that they have change so that they can improve the quality of life after being diagnosed with diabetes.

“*I have changed my lifestyle by having less family functions*, *no alcohol*, *no grog*, *no smoking so that my diabetes is controlled*. *And when I go to functions*, *I do not overeat and return home early*.*” (62-year-old Female Patient M10)*

Most participants do not exercise and only walk for minutes. Doing household chores is the only physical activity performed.

*“I only go for 10mintues walk in a day and do my household work*. *I don’t run as I have lot of house work to do and when I run*, *my knee starts to pain*.*” (71-year-old Female Patient M19)*

*Monitoring of blood sugar level as self-care management*. Most participants have blood sugar monitoring machine at home, however only some measure the sugar level daily before taking the medication.

“*I do have my blood sugar machine*, *but I do not check every time because it is very painful*. *My fingers are all hurt*. *Only once I week when I have eaten a lot then I ask my son to check”*. *(57-year-old Female Patient M28)*

Only one participant (n- 1) responded that he checks his sugar daily to see if the sugar is controlled after performing diabetes self-care management.

“*Every day I check my sugar to know my sugar reading*. *I also do that to know if my food and medication is helpful to me*.*” (40-year-old Male Patient M7)*

*Compliance to medication as self-care management*. Most of the patients are complaint to medication and trust that medications work to control sugar level.

*“I always take my medications on time because it really works on my body*. *Never mind my sugar level is good or bad when I check*, *I still take my medications*. *My children always ask me if I have taken my medication or not*. *Before I used to forget*, *and my sugar level gets high*. *Now I never forget*.*” (62-yeal-old Female*, *Patient M10)*

Few participants have also said that due to age and stress, she forgets to take her medications some times. Sometimes when she starts having signs of high sugar, then she remembers to take medication.

“*There were many times that I forgot to take my medication because I forgot about it*. *When my children notice that I haven’t taken the medication*, *then they remind me to take*. *Most times when I become very thirty or have dizziness then I remember that I did not take my medications*.*” (71-year-old Female Patient M11)*

*Use of herbal medications*. There were only few participants (n-4) who believed in herbal medication and consumes them to control sugar level.

*“I take leaves and drink to control my sugar*. *When I go to my village*, *then the villagers usually give me leaves to drink*.*” (52-year-old Male Patient M30)*

The participants also said that there is no harm following both treatments, as both help to control sugar.

“*I follow both things*. *I take medication given from the hospital and I also take herbal drinks as both things help me*. *My main aim here is that I do not want my sugar level to be high*, *so if herbal medicine is helping me then I will take it*.*” (57-year-old Female Patient M28)*

#### Health service delivery

The theme health service delivery had three sub-themes, service provided at health facilities, staff attitude influencing self-care management and attitudes toward conditions/health care environment at health facilities.

*Service provided at health facilities*. Most of the participants were happy with the service that was provided in the health centers.

“*The service here is very good*. *There is flow in the clinics as it is first come first service*. *Since I am not rich*, *I am grateful that everything here is free*, *only sometimes I have to buy medications when it is not in the health center pharmacy*.*” (71-year-old Male Patient M11)*

There were some participants who had problems with the service provided especially when they have appointment time.

“*At times I am disappointed with the service provided*. *As I am a working lady and I asked for 10am clinic which I was given*. *But on the clinic day I came little bit earlier and I was told I cannot be seen 30mintues earlier even it is empty*.*” (55-year-old Female Patient M3)*

*Staff attitude influencing self-care management*. Lot of participants have responded that the staffs are very good and polite.

“*The staffs here are so good*. *They are so polite when we go to them*. *They try their best to speak in the language that I can understand well*. *The doctor really takes time to explain my blood result*.*” (50-year-old Male Patient M1)*

Some participants have said, some staffs are so rude at times and get angry when sugar level is high.

*“Staffs are rude to me especially when my sugar level is very high*. *They ask very rudely what I eat and did I take my medications*. *And when I miss my clinic date*, *they do not want to see and make me sit for long and wait*.*” (56-year-old Male Patient M4)*

*Attitudes toward conditions/health care environment at health facilities*. Most of the participants were not happy we the waiting area space and ventilation.

“*The waiting area is very small for the patients to wait*. *Even it is very hard to breath when crowd comes at once*. *Sometimes there is not enough benches for us to sit*, *so have to squeeze in to sit*.*” (63-year-old Female Patient M5)*

Another participant also said, there is not enough ventilation and it is very hot at times with very close sitting.

“*It becomes very hot when it is full and there is no good air flow*. *When I have to wait for long*, *I feel like fainting*. *I have to sit very close to the next patient and I am scared*, *I might catch some disease*.*” (55-year-old Female Patient M17)*

#### Challenges and barriers faced by patients to perform self-care management

There are four sub-themes in challenges and barriers; socioeconomic status, family support, schedule for clinic date when working and strong family history of diabetes.

*Socioeconomic status affecting self-care management*. Most of the participant have said due to poor socioeconomic status they are not able to afford food that is right for diabetes.

“*I work as a cleaner and do not get much pay*. *Whatever I earn*, *I have feed my other family members as I am a widower*. *I mostly relay in foods like potatoes*, *normal rice and vegetables available at home as it is either free or cheap*. *I know potatoes and rice makes my sugar high*, *but I have to eat in order to survive*.*” (55-year-old Female Patient M3)*

Some participants have backyard gardens to support them, but it is not enough for daily food.

“*I have my garden at home and my family eats from there*, *but it is not enough to feed my whole family*. *Sometimes I sell from the garden then I buy other foods*. *So*, *I eat whatever is available*.*” (57-year-old Female Patient M28)*

*Family support influencing self-care management*. Most of the participants have poor family support, so eventually end up staying alone and utilizing the available money to buy the basic needs.

“*I my children are working but they stay with their own family*, *ever since my husband passed away*, *I stay alone with assistance from social welfare and have little money to use*.*” (61-year-old Female Patient M26)*

Some participants have said that they stay with family but have food that is good to eat for diabetes.

“*I prepare food for myself and the other family members prepare their own food*. *I use my pension money to buy my own food and my family has no problem*. *They support me to bring me for clinic*, *help me to exercise and remind me to take my medications*.*” (64-year-old Female Patient M21)*

*Schedule clinic for working patients*. All the working participants find it difficult to attend to clinic as they have to take sick leave.

*“Whenever I have clinic*, *I have to take sick leave so that I can attend the clinic properly*. *Before when I tell my company*, *they only give 1 hour to attend and return back to work*. *It is very difficult as the waiting time to be seen and then the waiting time in pharmacy is also long*, *get my pay gets detected*.*” (62-year-old Female Patient M10)*

Another participant also said, she always does not get leave to attend to clinic on weekdays.

“*I try my best to not to miss work but I have to due to work*. *Missing work affects my performance at work*. *Sometimes I have to miss my clinic date and get seen GOPD just to save my work*.*” (52-year-old Male Patient M18)*

#### Recommendations to improve diabetes self-care management

There are three sub-themes under this theme; flexible hours for working patients; quality of life; increase waiting area.

*Flexible hours for working patients*. All the working participants have suggested they can be give flexible hours to seen and separate from other patients.

“*I would like to suggest if working patients be seen early in the morning before 8am or after 4*.*30pm so that my working hours are not affected and not to rush*. *Sometimes my concentration is not on the advice given by doctors and nurses*, *but it’s that I am getting late to go back to work*.*” (50-year-old Male Patient M1)*

*Quality of life*. Most participants have recommended to include family members during clinic times.

*“It will be good if my family members are also included during counselling times so that they can help me*. *There can also know which foods to prepare for me*.*” (70-year-old Female Patient M6)*

Few participants have suggested to include counsellors to encourage them to perform self-care management and also to improve mental health.

“*There should be counsellors available to help me to do better in performing self-care management*. *it will also help me to think positive so that I can do things better in life*.*” (65-year-old- Male Patient M27)*

*Expansion of waiting rooms to create space and prevent congestion as patients wait for services*. Most participants have suggested there is a need to expand space for waiting area.

*“More space is needed for us to wait*, *and more benches are also needed to sit*. *When we come*, *we have to sit closely to another patient*. *It can also spread disease like cough*.*” (55-year-old Female Patient M17)*

Some participants have also suggested the health center should be renovated to make easier during raining weather. There should be more ventilation and more space available to sit.

“*There should be more waiting area and the whole health center should be renovated*. *During raining weather*, *it is very hard as the benches outside near entrance gets wet*. *The waiting area inside becomes too crowded*.*” (58-year-old Male Patient M25)*

## Discussion

The aim of this study was to know the perception of T2DM patients on diabetic self-care management in Central Division, Fiji. The study’s findings showed the knowledge of T2DM patients, the attitude and behavior of patients towards T2DM, the socio-economic status of patients and how it affects T2DM patients and the psychological status of patients.

### Discussion on the themes from the study findings

#### Knowledge of patients’ on T2DM self-care management

Participants in this study showed they have good knowledge on T2DM self-care management. All the participants knew the foods they should take; the medications they have to take, and some exercise they have to do. Participants also know the names of all the medication they take and how to keep them save from wound and cuts. Very few participants knew that at least 30minutes of exercise daily is needed. This study has found that knowledge is necessary for T2DM patients to make changes in their lifestyle. It is important for patients to continue gaining more knowledge about T2DM and self-care management according to the current socioeconomic status and the stress level. Stress management, accepting T2DM as a disease and coping with the disease are some new factors found that lacks in patients and they have limited knowledge about it. The new thing found in this study is that patients know the word exercise in self-care management, but do not know which exercise is effective enough to maintain a good sugar level. Most patients consider walk is a very good, but they are not explained properly how intense the walk should be in order to burn the calories. Evidence from this study has supported previous studies that diet, medication and physical activity are basic knowledge for patients [25, 26]. Exercise is considered the most important component to maintain good physiological and psychological part of health [27].

#### Attitude and behavior of patients towards self-care management

This study found out that patients are very compliant with medication and believe it is the most important part in T2DM self-care management. Most patients only go for 10mintues of walk and other patients know that doing household work is enough exercise for the day. The findings demonstrate patients are not aware of the importance of physical activity compared to other self-care management behaviors. Most patients do not go for exercise in gyms as it is too expensive and location is far, difficulty to incorporate exercise to daily lives due to work and some patients are culture sensitive [28]. Some of the patients’ belief in both; herbal medications and also hospital medications. This study has also shown that most patients do not take sugar or take very less amount of sugar. This study has also found the least performed self-care management behavior was foot care. Hardly any patient checks foot and cut the nails. The only time foot is checked is when it starts getting infected. In Jordanian patients, foot care is one of the most practiced self-care management due to religious belief and find it a very practical belief in taking care of own selves [27].

Patients only know compliance to medication is more important than any other self-care management and belief that two things are very important in diabetes care; adherence to medication and self-care management behavior [29]. To debate, a study done in Kurdistan, showed patients are non-compliant to medications and do not trust health professional on the medications they are prescribed. The participants’ belief on their own treatment and do not belief on scientific ideas of the health team [30]. This study supports gaining new knowledge from new research and advanced management can help bring change in attitude and behavior of T2DM patients. This study has shown its just patients less of interest to perform self-care management due to loss of interest, stress and depression. The study also showed patients have gained good knowledge from HCWs, however, they are not able to put it to practice due to lack of motivation from within self. Similar studies have shown, the more knowledge the patients receive in every clinic, the more their behavior and attitude changes towards T2DM self-care management [25, 31].

#### Socio-economic status and social life of the patients

This study has shown that most patients have socio-economic status that can only support basic needs and they have to compromise their diet plan. They eat what is available to survive and have no choice for diabetic diet. Most of the patients stay with family members and eat whatever they are provided with. Patients have also pointed out some family members have reluctance to modify family diet due to lack of understanding. Socio economic status of patients in this study was determined by the source of income and the income range. Most of the patients rely on social welfare assistance from the government, some of patients are using their retired pension and only few patients are still working. Low socio-economic status and T2DM complication is directly related among young adults diagnosed with T2DM. Socioeconomic status is one of the causes for T2DM patients to get T2DM complications such as retinopathy and nephropathy (Funakoshi et al., 2017). The disadvantaged socio economic patients tend to have higher obesity incidence due to high fatty food, high carbohydrate food and less fruits and vegetables [32]. Due to low socioeconomic status, T2DM patients consume poor quality diet which increases their glycemic level. Low, middle and high socioeconomic are all risk factors for T2DM patients. Patients with low socio economic status are not only affected by poor quality of diet, but they are mentally stressed leading to decreased physical activity, thus increasing the risk of T2DM complications [33].

Family and social life of patients in the study has both influential effect and discouraging effect. The study has also shown that due to very friendly and social environment of families and communities, some patients get influenced and fail to follow proper dietary pattern of diabetes. Most patients crave to eat traditional foods some of which contain lot of sugar which then becomes a problem in few days’ time. Some family members are so considerate about the sugar level of the family members that they prepare special diet for the family members suffering from diabetes in social gatherings. This study has also found that family plays a very important part in improving the quality of life of the patients. The family members encourage and remind the patients to take medications, go for exercise and eat healthy [34, 35].

#### Psychological impact

This study has also shown that most patients are so stressed about their sugar level that they become helpless. Most of them have diabetes because it is in their genetics and even after trying their best with self-care management, they are not able to control their sugar level. Most patients have also said they were in denial stage when first they were told they have diabetes. Due to beginning in denial stage, there were lot of complications of diabetes noted. The interesting part discovered in this study is nearly all patients are stressed due to T2DM, but there is no psychosocial treatment or formal counselling given to patients. Patients are carrying all the burden of the disease themselves and are stressed. There was no guideline or no intervention plan to treat and monitor stressed patients. Due to psychological and psychiatric issues, T2DM patients get associated with its complications such as poor dietary pattern, emotional stress, fear of hypoglycemia, poor compliance to medication and exercise [36]. This study showed emotional stress in T2DM patients when they first came to know about the disease and having reactions of shock, denial, anger and anxiety. According to the patients, as the disease progressed over the years, diabetes distress of patients is more visible in patients when they are getting frustrated as they are compelled to be compliant to self-care management and differential of food in family. Patients have also revealed phobia of needles, insulin treatment and risk of experiencing hypoglycemia when taking medications. A similar finding was seen in a study that the emotions expressed by patients are part of the emotional stages which can lead to psychological disorder if not treated earlier [36]. There are some patients with the fear of stigma especially when they are in a social gathering. Patients are scared if family or friends do not come to know that they have diabetes, hence eat whatever is served and pretend they are normal. Some patients have also expressed that they do not have confidence to eat medications in front of others. Similar study has shown patients are stigmatized with feeling of fear, blame, embarrassment, guilt and low self-esteem. These negative emotions can put patients into depression resulting in high level of blood glucose readings [37]. This study has also shown self-care management is a habit that should come from within a person. If the mindset of patients is not focused to achieve the desired goal, then no education or counselling can help. It is just the patient himself or herself who can bring the change.

#### Study limitations

This study had few limitations. Firstly, the result of this study is not representative of all T2DM patients in whole of Central division, Fiji. Secondly, the study sample from this study was from a small geographic location. It is not a true representative for whole of Fiji. Thirdly, all the participants were informed during the consent process that their interviews will be recorded, which may have influenced them to respond differently from the normal circumstances. Fourthly, the research was limited to citizens of Fiji only, thus the immigrants’ experiences are not included in the study.

## Conclusion

T2DM self-care management has been globally recognized as one of the most effective way to help improve and live a better quality of life. Patients in this study knew about T2DM self-care management and practiced self-care management to improve quality of life and remarked that they made great effort to attend SOPD clinic in health centers. This study highlighted that T2DM patients were also psychologically affected which hindered them to perform T2DM self-care management. The lifestyle of patients in Fiji had huge impact on self-care management on T2DM patients. To add-on, the attitude and behavior of T2DM and the socioeconomic status of patient influences the self-care management practices. Like all other countries, patients in Fiji have challenges when it comes to family support, health services, and family history of T2DM. These challenges have affected T2DM patients’ quality of life by having more complications such as poor vision, amputations and stress. The findings of this research identified that there are gaps that exists and potentials for improvement on ownership of own health which can be achieved by strengthen of knowledge of patients on the importance of performing T2DM self-care management from the first education class. It is recommended for health facilities improve the space provided in the waiting area with good ventilation. There is a need to collaborate more with other multidisciplinary team such as counselors and agriculture department.
